# Retrospective longitudinal analysis of blood microRNA-7-5p as a possible progression biomarker in people with Parkinson’s disease

**DOI:** 10.3389/fnins.2026.1784013

**Published:** 2026-04-14

**Authors:** Shayan Abdollah Zadegan, Corrine Hutchinson, Chiamaka Onuigbo, Juan D. Martinez-Lemus, Laura Talbot, E. Jeffrey Metter, Marie-Francoise Doursout, Emily Tharp, Jessika Suescun, Mohammad Shahnawaz, Timothy M. Ellmore, Mya Schiess, Christopher Adams

**Affiliations:** 1 Department of Neurology, The University of Tennessee Health Science Center, Memphis, TN, United States; 2 Department of Neurology, McGovern Medical School, University of Texas Health Science Center, Houston, TX, United States; 3 Department of Anesthesiology, McGovern Medical School, University of Texas Health Science Center, Houston, TX, United States; 4 Department of Neurology, Queensland University of Technology, Brisbane, QLD, Australia; 5 Department of Psychology, The City College of New York, New York, NY, United States

**Keywords:** alpha-synuclein, biomarkers, microRNA, neurofilament light chain, NLRP3 inflammasome, Parkinson’s disease

## Abstract

**Background:**

MicroRNA-7-5p (miR-7-5p) may play a neuroprotective role in people with Parkinson’s disease (PwP), as it has been found to regulate *α*-synuclein (α-syn) and the NLRP3 inflammasome in animal models of Parkinson’s disease (PD).

**Objective:**

The study aimed to investigate the use of miR-7-5p as a potential biomarker for disease progression in PwP by correlating it with time, clinical measures, and neurofilament light chain (NfL).

**Methods:**

We performed a longitudinal retrospective analysis of blood miR-7-5p levels in 303 *de novo* PwP and 159 healthy controls (HCs) from the Parkinson’s Progression Markers Initiative (PPMI) cohort. In PwP, a linear mixed-effects model was used to examine the association between miR-7-5p levels and time in the study. In addition, linear mixed-effects models were used to examine the associations between longitudinal changes in miR-7-5p and scores on the Movement Disorder Society Unified Parkinson’s Disease Rating Scale (MDS-UPDRS), both for motor and total scores, as well as serum NfL levels. These models were also used to compare the associations between changes in miR-7-5p, time in the study, and NfL levels in both PwP and HCs.

**Results:**

miR-7-5p levels decreased more rapidly in PwP compared to HCs (*p* = 0.02). In PwP, miR-7-5p levels correlated with time in the study (*p* < 0.001) and with changes in the MDS-UPDRS motor (*p* = 0.007) and total scores (*p* = 0.01). However, when time in study was taken into account, the correlations were no longer significant. Additionally, miR-7-5p levels decreased longitudinally as NfL levels increased in PwP (*p* = 0.03), but this did not remain significant when time in the study was considered.

**Conclusion:**

This pattern suggests that miR-7-5p may reflect both upstream pathogenic mechanisms and downstream neuroaxonal damage. However, the loss of significance when time in the study was included in the model indicates that the changes may reflect parallel degenerative processes involving NfL and miR-7-5p. This study is novel in that it demonstrates a correlation between miR-7-5p levels, clinical severity, and NfL levels.

## Introduction

Parkinson’s disease (PD) is a progressive neurodegenerative disorder characterized by multifactorial and complex underlying mechanisms. Alpha-synuclein (*α*-syn) accumulates and forms toxic aggregates in different regions of the brain, particularly in the substantia nigra and striatum. These aggregates trigger a cascade of negative consequences, including oxidative stress, mitochondrial dysfunction, immune dysregulation, and apoptosis. These factors collectively contribute to the progressive degeneration of dopaminergic neurons (Rocha et al., 2018). The complexity of PD has made it challenging to establish a definitive biomarker for diagnosis or disease progression. Currently, the gold standard for diagnosis relies on patient history and clinical assessment, which can sometimes result in delays or errors in diagnosis. While advanced imaging and *α*-syn testing are currently used in practice, they lack the specificity needed to reliably distinguish PD from other atypical parkinsonian syndromes or synucleinopathies. Moreover, emerging biomarkers, such as neurofilament light chain (NfL), tau protein, and Aβ42, may help monitor disease progression, but they are also limited by their lack of specificity. Identifying reliable markers of disease progression is essential not only to improve diagnostic accuracy and monitor therapeutic responses but also to support the development and evaluation of disease-modifying treatments (Arya et al., 2024).

MicroRNAs (miRNAs) are non-coding RNAs that play a key role in regulating protein expression, primarily by inhibiting mRNA translation or facilitating mRNA or protein degradation. miRNAs have emerged as promising candidates for biomarkers of diagnosis and disease progression in people with Parkinson’s disease (PwP). One notable miRNA, microRNA-7-5p (miR-7-5p), was found to be decreased in the substantia nigra of PwP compared to healthy controls (HCs; McMillan et al., 2017). This miRNA has gained attention due to its role in regulating the NLRP3 inflammasome and *α*-syn aggregation in animal models of PD (Doxakis, 2010; Junn et al., 2009; Choi et al., 2018; Zhang et al., 2021). Activation of the NLRP3 inflammasome leads to sustained microglial activation, contributing to a pro-inflammatory state. miR-7-5p inhibits the NLRP3 inflammasome, resulting in an anti-inflammatory effect and a reduction in the propagation of *α*-syn (Lema Tome et al., 2013; Zhou et al., 2016). In addition, miR-7-5p has been shown to promote the degradation of extracellular *α*-syn fibrils (Choi et al., 2018; Zhang et al., 2021), which helps to reduce their pathological accumulation. Furthermore, miR-7-5p can bind to the 3′ untranslated region (UTR) side of the SCNA gene (Shaheen et al., 2024), decreasing mRNA stability and limiting α-syn proliferation. Finally, miR-7-5p is downregulated by atrazine and rotenone, which are pesticides that damage the substantia nigra in mouse models of PD. This suggests that miR-7-5p may be susceptible to disruption by other environmental toxins implicated in the pathogenesis of PD (Li et al., 2019; Horst et al., 2018).

While previous studies have suggested that miR-7-5p could serve as a clinically useful biomarker in PwP (Citterio et al., 2023; Ardashirova et al., 2022), both independently and in conjunction with ancillary data, its utility as a marker for disease progression in PwP has only been assessed in one previous study (Kern et al., 2021). Kern et al. found that miR-7-5p levels differed significantly between 36 and 12 months, as well as between 36 months and the baseline (Kern et al., 2021). To the best of our knowledge, no clinical study has examined whether changes in miR-7-5p levels correlate with validated measures of disease severity or progression in PwP. To better assess its potential as a biomarker for disease progression, our study sought to determine whether longitudinal changes in miR-7-5p levels in PwP correlate with changes in established clinical and blood measures of disease progression, using HCs for comparison.

## Methods

### Study data

Data from participants were obtained from the Parkinson’s Progression Markers Initiative (PPMI), an ongoing longitudinal observational study that serves as a biobank of PwP and HCs from North America and Europe. The goal of the PPMI is to encourage a collaborative effort among researchers to identify markers of PD risk, diagnosis, and progression. Further details on inclusion and exclusion criteria for each cohort can be found on their website: www.ppmi-info.org/study-design. The PPMI studies received approval from the institutional review boards at each participating site, and written informed consent was obtained from participants before enrollment. Local IRB approval was not required as all personal health information had been removed from the data.

### Study population

Permission was obtained through the PPMI data portal at the Laboratory of Neuroimaging, University of Southern California. The data were accessed on 17 September 2023 and the analysis for this study included participants enrolled between 1 February 2011 and 1 April 2016. This study cohort included PwP and HCs. According to PPMI guidelines, the inclusion criteria for PwP included positive dopamine transporter (DaT) imaging findings, clinical features supporting a diagnosis of PD, no prior exposure to dopaminergic treatment, a disease duration of no more than 2 years since the initial diagnosis, and a Hoehn and Yahr (H&Y) score of 3 or lower. In addition, this study included only PwP aged 50 years or older. This age cutoff was used to reduce the likelihood of including cases with unknown genetic causes of PD that may not be detected by current available genetic testing. The inclusion criteria for HCs were adults over 30 years of age with no neurological disorders and no first-degree relatives diagnosed with PD. Furthermore, HCs could not carry GBA or LRRK2 mutations.

### Clinical and biomarker assessments

To evaluate the potential of miR-7-5p as a biomarker, the following variables were collected: Age, sex, PD medication status, NfL, and Movement Disorder Society Unified PD Rating Scale (MDS-UPDRS) total and motor scores.

NfL data were obtained directly from the PPMI database. Serum was isolated from blood for NfL measurements (pg/mL). NfL levels were measured in duplicate using the Simoa NF-light Advantage Kit (Quanterix, Lexington, MA) on a fully automated Simoa HD-1 Analyzer (Mollenhauer et al., 2020). The natural log of NfL (log[NfL]) was used for normalization in the analyses.

miRNA data were also obtained from the PPMI database. Whole blood was collected in PAXgene tubes and sequenced using an Illumina NovaSeq 6,000 platform with Bioo Scientific smRNA library preparation. Expression files processed by the PPMI were provided as raw counts and reads per million (RPM). For miRNAs, data were normalized to reads per million mapped to miRNAs (RPMMM), and miRNAs with zero expression (RPM = 0) in more than 50% of samples were excluded. The natural log of miR-7-5p (log[miR-7-5p]) was used for normalization in the analyses. Full details of sequencing and preprocessing are available in the PPMI documentation and in the methods of a previous study (Kern et al., 2021). While RNA sequencing was conducted in two phases, miRNA sequencing was performed in the first phase (PPMI project 133).

### Statistical analysis

Baseline demographic characteristics were compared between the PwP and HC groups using a chi-squared test for sex distribution. Tests for normality and heteroscedasticity were conducted on the data. Since most variables, except for NfL, did not follow a normal distribution, medians and interquartile ranges were used as descriptors of central tendency. In addition, the Mann–Whitney U test was used for age, MDS-UPDRS motor scores, MDS-UPDRS total scores, and log[miR-7-5p] levels. A *t*-test was used for log[NfL] levels. The PPMI cohort was not age- or sex-matched to maintain statistical power for the analysis. As a result, age and sex were used as covariates instead during regression modeling. All participants included in the set were followed for 36 months. The time points analyzed included baseline (*N* = 457), 6 months (*N* = 301), 12 months (*N* = 379), 24 months (*N* = 364), and 36 months (*N* = 352).

Longitudinal changes in miR-7-5p were analyzed using linear mixed-effects models. Log[miR-7-5p] levels were modeled as a function of time in the study, diagnostic group, and their interaction as fixed effects. Baseline age and sex were included as covariates. The interaction term was included to assess whether the trajectory of miR-7-5p differed between HCs and PwP over time. To determine whether comorbidities associated with decreased miR-7-5p levels, such as thyroid cancer, hypertension, atrial fibrillation, type 2 diabetes, or COVID-19, might contribute to the decrease in miR-7-5p observed in HCs, mixed-effects linear models were used. Similar mixed-effect models were used to examine associations between log[miR-7-5p] and time in the study, clinical measures (MDS-UPDRS motor and total scores), and log[NfL] in PwP. Age, medication status, and sex were used as fixed effects. Clinical measures and log[NfL] levels were modeled with and without time as a fixed effect to determine whether the correlations were independent of time. Medication status was coded as a binary factor indicating the time point at which medications were started. A combined model including both HCs and PwP was used to assess the interaction between diagnostic group and log[Nfl] on log[miR-7-5p]. All linear mixed-effects models were fitted using the R “lmerTest” package. Data visualization was performed using the “ggplot2” package.

Since linear mixed-effects models were applied, participants with incomplete longitudinal biomarker data were included, under the missing-at-random (MAR) assumption. Therefore, individuals with missing miR-7-5p values at certain time points were retained in the analysis. *p*-values were adjusted for multiple comparisons using the false discovery rate (FDR) method in R. A *p*-value of < 0.05 was considered statistically significant.

## Results

### Baseline characteristics

**Figure 1:**
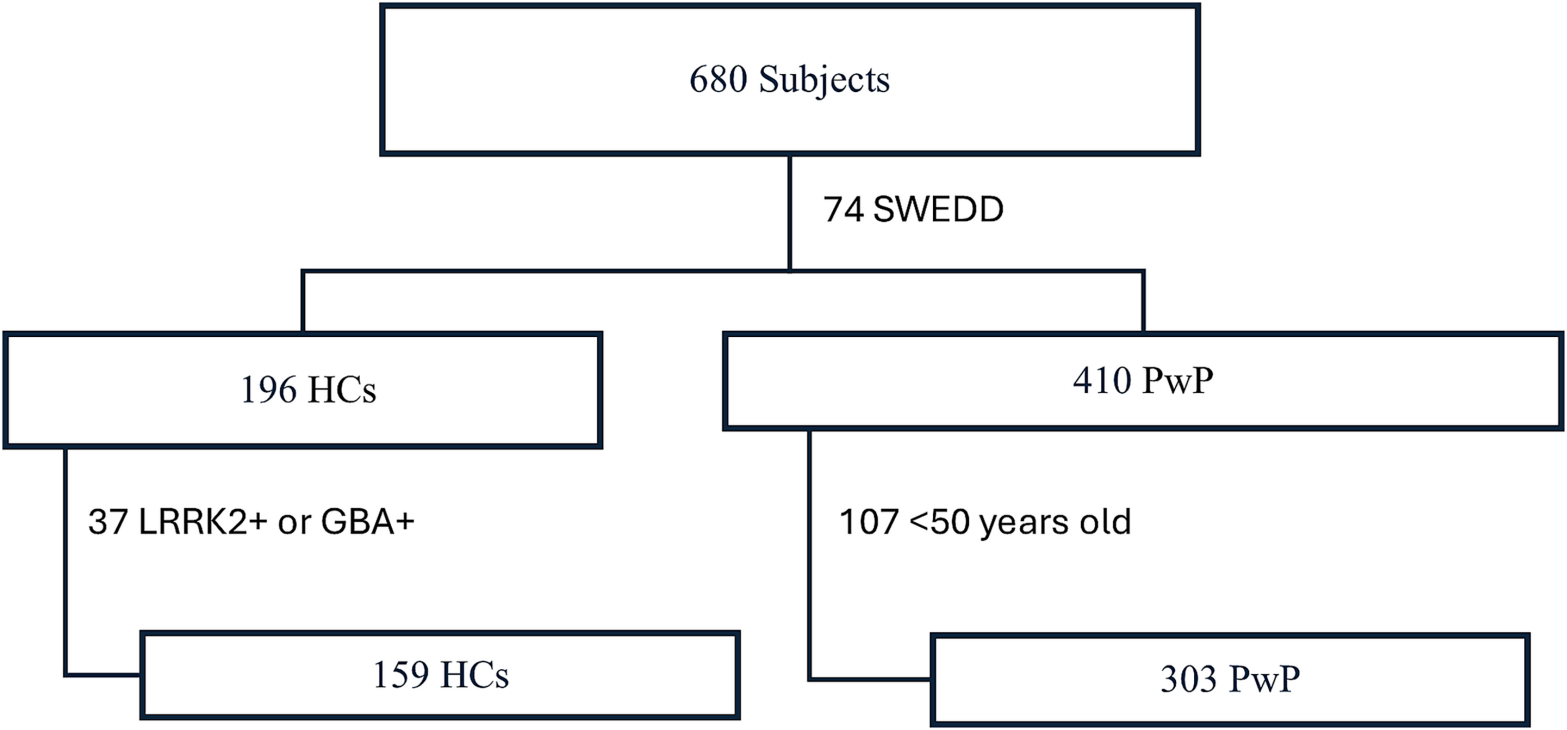
Flow diagram of participants included in the study. SWEDD, Scans without evidence of dopaminergic deficit; HC, healthy control; PwP, people with Parkinson’s disease.

Baseline characteristics appeared similar across groups, including sex distribution (Table 1). Data for 196 HCs and 410 PwP were obtained from the PPMI database. A total of 74 participants were excluded because they had DaT scans without evidence of dopaminergic Deficit (SWEDD). An additional 107 PwP were excluded for being younger than 50 years. There were no genetic forms of PD in this cohort, as a separate PPMI cohort exists for genetic forms of Parkinson’s disease. In addition, 37 HCs were excluded due to LRRK2 + or GBA + status. The final analysis included 303 PwP and 159 HCs (Figure 1).

**Table 1:** Baseline demographic characteristics.

Parameter	Parkinson’s disease	Healthy control	*p*-value
N	303	159	NA
Male/Female (ratio)	199/104(1.91)	106/53 (2)	0.87
Age, median (IQR)	64.76 (58.99–69.76)	63.99 (57.34–69.33)	0.22
Disease duration (yrs), median (IQR)	0.33 (0.21–0.66)	NA	NA
MDS-UPDRS motor, median (IQR)	20 (15–26)	0 (0–2)	<0.001
MDS-UPDRS total, median (IQR)	31 (23–40)	3 (1.5–7)	<0.001
log[miR-7-5p], median (IQR)	3.68 (3.21–4.13)	3.72 (3.21–4.07)	0.78
log[NfL], mean (SD)	2.55 (0.44)	2.39 (0.44)	<0.001

Data are presented as median (interquartile range, IQR). Group comparisons were performed using the Mann–Whitney U test for age, MDS-UPDRS motor and total scores, and log[miR-7-5p] levels. A t-test was used for log[NfL] levels. A chi-squared test was used for sex. A p-value of <0.05 was considered statistically significant. MDS-UPDRS, Movement Disorder Society Unified Parkinson’s Disease Rating Scale; NfL, Neurofilament light chain.

PwP had a median age of 64.76 years and a median disease duration of 0.33 years. They had a median MDS-UPDRS motor score of 20 and a total score of 31 (Table 1). In contrast, HCs had a median age of 63.50 years, a median MDS-UPDRS motor score of 1.39, and a total score of 4.92 (Table 1). As expected, PwP had significantly higher MDS-UPDRS motor scores (*p* < 0.001) and total scores (*p* < 0.001) compared to HCs (Table 1). There was no significant difference between PwP and HCs regarding male/female distribution, age, or log[miR-7-5p] levels (Table 1). However, log[NfL] levels were significantly higher in PwP compared to HCs (*p* < 0.001; Table 1).

### Mixed-effects model of longitudinal changes in miR-7-5p

**Figure 2:**
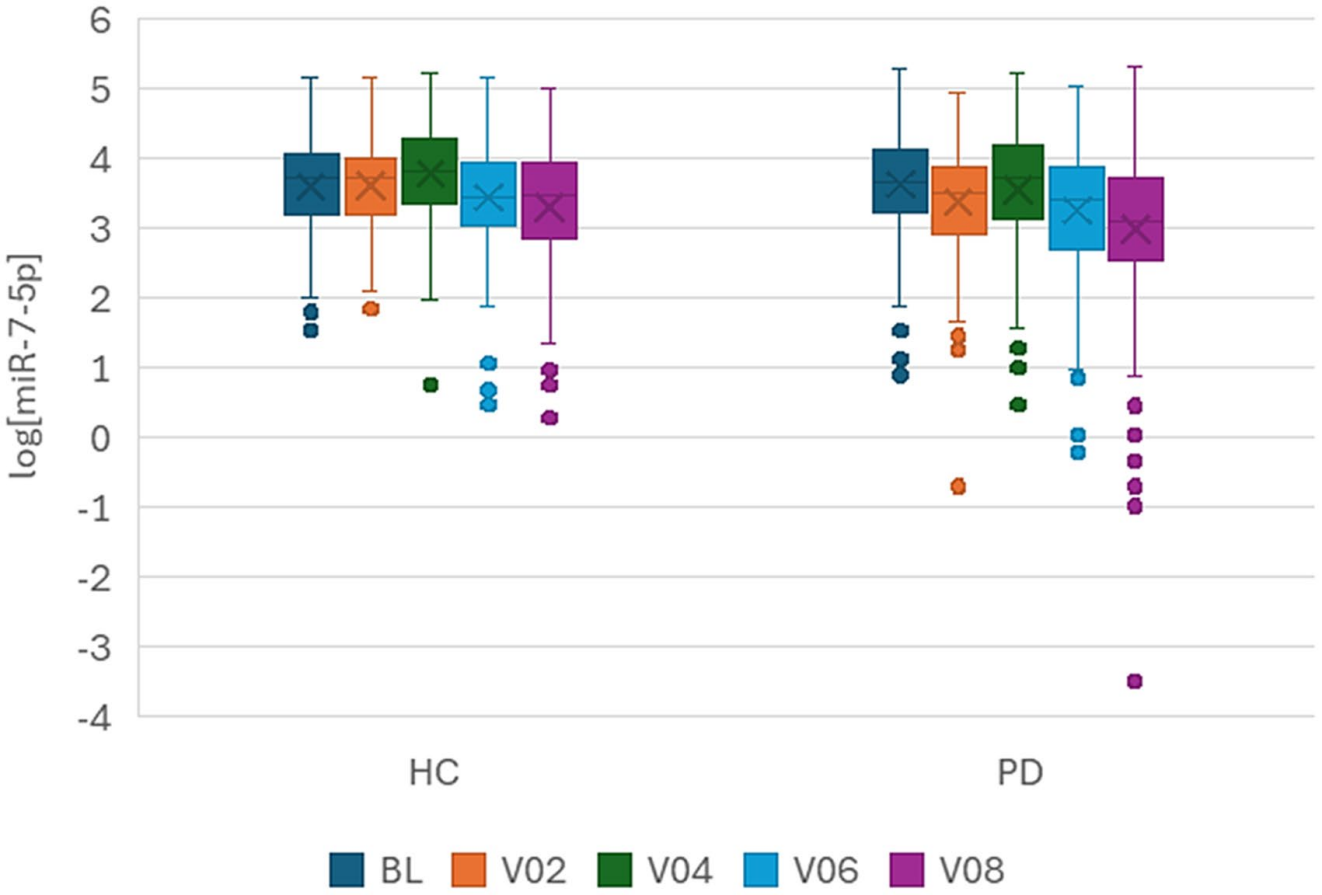
Boxplot of log[miR-7-5p] at baseline (BL, dark blue), 6 months (V02, orange), 12 months (V04, green), 24 months (V06, light blue), and 36 months (V08, purple).

**Figure 3:**
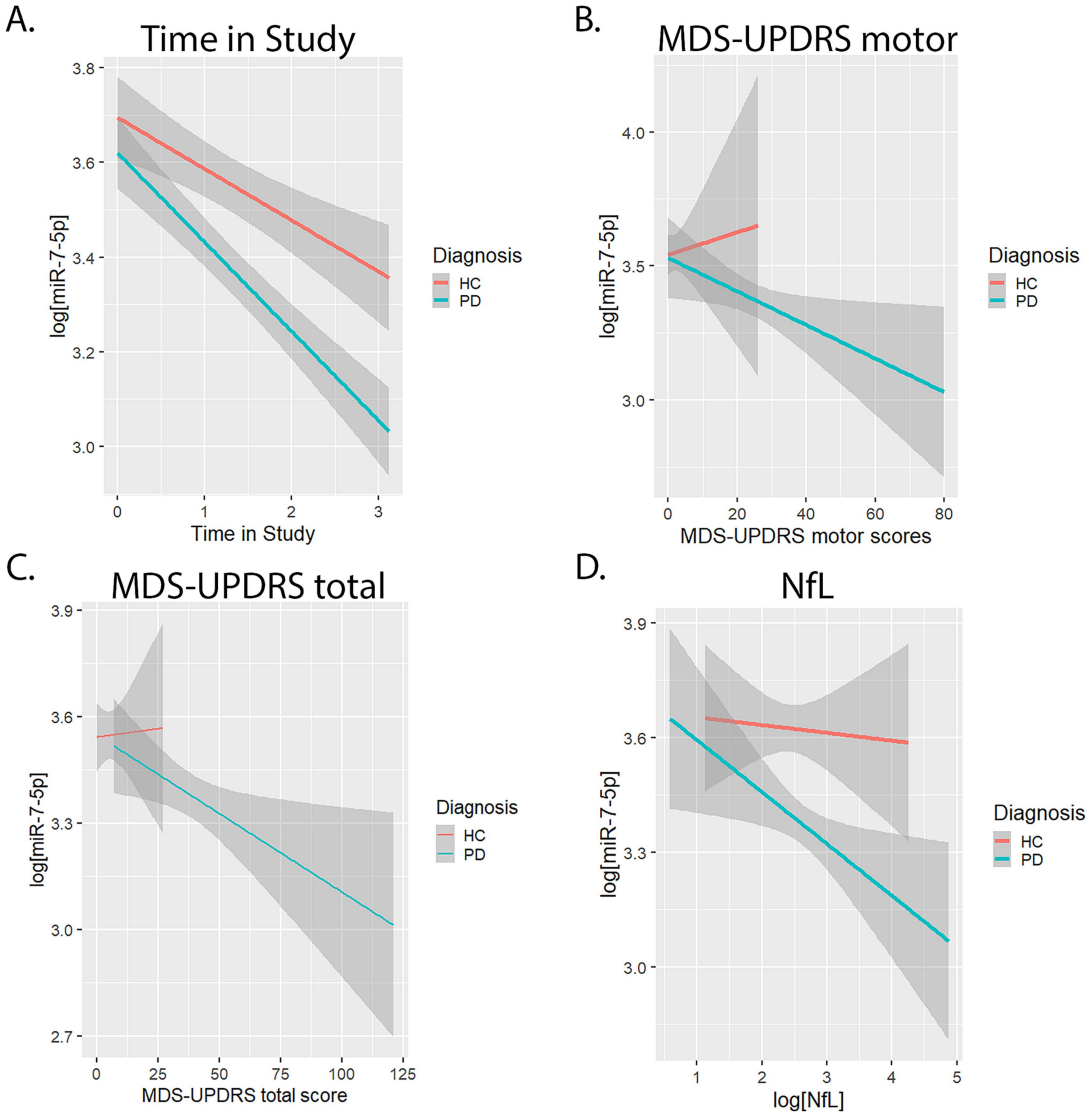
Mixed-effects linear model plots of various measures vs. miR-7-5p, using participant ID as a clustering variable. **(A)** Linear plots of log[miR-7-5p] (reads per million [rpm]) vs. time in the study for HCs (red line) and PwP (blue line). **(B)** Linear plots of log[miR-7-5p] (rpm) vs. MDS-UPDRS motor scores for HCs (red line) and PwP (blue line). **(C)** Linear plots of log[miR-7-5p] (rpm) vs. MDS-UPDRS total scores for HCs (red line) and PwP (blue line). **(D)** Linear plots of log[miR-7-5p] (rpm) vs. log[NfL] (pg/mL) for HCs (red line) and PwP (blue line).

A boxplot showed that miR-7-5p decreased over time in both HCs and PwP (Figure 2). Diagnosis, sex, age, and time in the study were included as fixed effects in a mixed-effects model. Participant ID was included as a clustering variable and a random effect to account for repeated measurements. While there was no main effect of diagnosis on log[miR-7-5p] levels, there was an interaction effect between diagnosis and time in the study (*p* = 0.02, FDR-adjusted). Figure 3A shows time in the study vs. log[miR-7-5p]. The results did not significantly change when time in the study was included as a random effect, indicating that the individual rates of change in miR-7-5p over time were similar within each group. Furthermore, the results did not change when random slopes were used.

In PwP, log[miR-7-5p] levels decreased longitudinally in a mixed-effects model including time in the study, PD medication status, sex, and age as fixed effects (*p* < 0.001, FDR-adjusted). Participant ID was used as a clustering variable and a random effect. Medication status did not significantly influence miR-7-5p levels. Similarly, in HCs, log[miR-7-5p] levels decreased longitudinally in a mixed-effects model including time in the study, sex, and age as fixed effects (Figure 3A). miR-7-5p decreased more rapidly in PwP than in HCs. While miR-7-5p decreased by 15.5% per year in PwP (*p* < 0.001, FDR-adjusted; Table 2), it decreased by 10% per year in HCs (*p* < 0.001).

**Table 2:** Linear mixed-effects models of various measures vs. miR-7-5p in PwP.

Parameter	β	Exp(β)	Standard Error	FDR-Adjusted *p*-value
Time in the Study (yr)	−0.184	0.836	0.02	<0.001
MDS-UPDRS motor	−0.008	0.992	0.003	0.01
MDS-UPDRS total	−0.007	0.993	0.002	0.006
log[NfL]	−0.18	0.914	0.08	0.153

miR-7-5p vs. time in the study was adjusted for sex, age, and medication status. miR-7-5p vs. MDS-UPDRS motor scores, MDS-UPDRS total scores, and log[NfL] were adjusted for sex and age. The time points analyzed included baseline (*N* = 457), 6 months (*N* = 301), 12 months (*N* = 379), 24 months (*N* = 364), and 36 months (*N* = 352). PwP, People with Parkinson’s disease; yr, year; MDS-UPDRS, Movement Disorder Society Unified Parkinson’s Disease Rating Scale; NfL, Neurofilament light chain; Exp(β), exponentiation of β; β, estimated change in the dependent variable for a one-unit change in the predictor variable.

### Mixed-effects models of miR-7-5p vs. clinical measures and NfL

When analyzing the correlation between clinical measures and log[miR-7-5p], MDS-UPDRS motor and total scores, sex, and age were included as fixed effects in the mixed-effects models. Time was not included in these models. Different mixed-effects models were used to examine the correlations between clinical measures or log[NfL] and log[miR-7-5p] in PwP. Analyses were performed with and without time in the models. Participant ID was used as a clustering variable and a random effect.

In a mixed-effects model, as log[miR-7-5p] levels decreased, MDS-UPDRS motor scores (*p* = 0.01, FDR-adjusted) and total scores (*p* = 0.006, FDR-adjusted) increased (Figures 3B,C; Table 2). For each unit increase in MDS-UPDRS motor and total scores, there was a 0.8 and 0.6% decrease in miR-7-5p levels, respectively (Figures 3B,C). However, when time in the study was included in the model, neither MDS-UPDRS motor scores nor total scores remained statistically significant. In HCs, miR-7-5p did not correlate with MDS-UPDRS motor or total scores (Figures 3B,C).

In PwP, log[miR-7-5p] levels significantly decreased longitudinally as log[NfL] levels increased, when baseline age and sex were included as covariates (*p* = 0.02, FDR-adjusted) (Figure 3D; Table 2). However, when time was added to the model, the relationship between log[miR-7-5p] and log[NfL] was no longer statistically significant. In HCs, a model including baseline age and sex as covariates showed no relationship between log[miR-7-5p] levels and log[NfL] levels (*p* = 0.61).

In a mixed-effects model, there was no significant interaction between diagnostic group and log[NfL] levels on log[miR-7-5p] levels (*p* = 0.153, FDR-adjusted). Log[NfL], diagnosis, sex, and baseline age were included as fixed effects in this mixed-effects model. Participant ID was used as a clustering variable and a random effect.

### Analysis of miR-7-5p levels in those with comorbidities associated with decreased miR-7-5p levels

In a mixed-effects model including time in the study, diagnosis, comorbidities associated with decreased miR-7-5p, sex, and baseline age, there remained a significant interaction between time in the study and diagnosis (*p* = 0.02).

In HCs, a mixed-effects model including time in the study, baseline age, sex, and comorbidities showed that time in the study was no longer significant (*p* = 0.07) (Table 3). In this model, miR-7-5p levels changed by 5% per year. There was a significant interaction between time in the study and comorbidities (*p* = 0.001).

**Table 3:** Linear mixed-effects models including comorbidities associated with decreased miR-7-5p vs. miR-7-5p.

Parameter	β	Exp(β)	Standard error	*p*-value
Time in the study for HCs (yr)	−0.05	0.947	0.03	0.07
Time in the study for PwP (yr)	−0.184	0.832	0.02	<0.001

In the linear mixed-effects models, miR-7-5p vs. time in the study for PwP or HCs, sex, baseline age, time in the study, and comorbidities associated with decreased miR-7-5p were included. HCs, Healthy controls; PwP, People with Parkinson’s disease; Exp(β), exponentiation of β; β, estimated change in the dependent variable for a one-unit change in the predictor variable.

In PwP, time in the study remained significant (*p* < 0.001) in a model including time in the study, baseline age, sex, and comorbidities (Table 3). In this model, miR-7-5p levels changed by 17% per year. There was no significant interaction between time in the study and comorbidities.

## Discussion

miR-7-5p is highly expressed in dopaminergic neurons in the substantia nigra and has been shown to protect dopaminergic neurons in MPTP and *α*-syn PD animal models. We explored mixed-effects models examining the relationship between peripheral blood log[miR-7-5p] levels, time, clinical severity, and log[NfL] serum levels. In PwP, log[miR-7-5p] decreased significantly as the disease progressed. In addition, the decline over time was greater in PwP than in HCs when sex and age were considered. While log[miR-7-5p] also decreased over time in HCs, the correlation was no longer significant when comorbid conditions associated with decreased miR-7-5p were included in the model. However, when comorbidities were included in a model for PwP, the correlation remained significant. In addition, miR-7-5p was inversely correlated with MDS-UPDRS motor and total scores in PwP when sex and age were considered. However, the correlation was no longer significant when time in the study was included in the model. Furthermore, miR-7-5p was inversely correlated with NfL in PwP but not in HCs. However, this correlation in PwP was no longer significant when time in the study was considered.

miR-7-5p regulates NLRP3 translation and inhibits its activation, which is likely an important component of PD pathogenesis. *α*-syn aggregation induces an inflammatory response through the production of interleukin-1β in a process that relies on the NLRP3 inflammasome (Pike et al., 2021). The NLRP3 inflammasome is highly expressed in microglia and oversees the initiation and perpetuation of the inflammatory cycle. Indeed, inhibition of the NLRP3 inflammasome has been shown to mitigate motor deficits, dopaminergic degeneration, and the accumulation of α-syn aggregates in PD animal models (Gordon et al., 2018). Furthermore, plasma from PwP and post-mortem brain tissue from PwP showed increased NLRP3 + cells, and its levels correlated with dopaminergic neuron loss (Anderson et al., 2021). Activation of the NLRP3 inflammasome has been found to be elevated in the plasma of PwP and correlates with both H&Y scores and MDS-UPDRS motor scores (Fan et al., 2020). Furthermore, NLRP3 inflammasome activation correlates with plasma α-syn (Fan et al., 2020). A polymorphism in NLRP3 is associated with a decreased risk of PD (von Herrmann et al., 2018). Recently, an NLRP3 inflammasome inhibitor was tested in a phase I clinical trial (Clarke et al., 2025). They found consistent reductions in NfL and in inflammatory molecules associated with PD. miR-7-5p may be an ideal biomarker for this study as it directly regulates the NLRP3 inflammasome. It can be postulated that miR-7-5p would decrease more slowly over time with the NLRP3 inflammasome inhibitor. However, this requires further study.

NfL, a critical component of the neuronal cytoskeleton structure, showed a strong negative correlation with miR-7-5p. In a landmark study by Mollenhauer et al., longitudinally evaluated NfL levels were shown to correlate with disease progression and traditional motor clinical assessments in PwP (Mollenhauer et al., 2020). Mollenhauer et al. reported an NfL increase of 3.8% per year in PwP, suggesting that disease progression is associated with increasing axonal damage, resulting in elevated serum NfL levels (Mollenhauer et al., 2020). This supports the inverse relationship with miR-7-5p observed in PwP but not in HCs. NfL provides information about disease progression in PwP by reflecting the intensity and extent of neuroaxonal damage. NfL levels in blood and CSF increase in proportion to ongoing axonal degeneration, regardless of the specific molecular trigger (Yuan and Nixon, 2021).

One limitation of NfL is its lack of specificity for PD, as levels are elevated in a variety of neurodegenerative conditions (Virata et al., 2024). Furthermore, accurate measurement of NfL requires access to specialized equipment, which requires special training to operate and maintain. Another limitation is that most neuron loss occurs prior to diagnosis, meaning that prodromal patients may have higher NfL levels than diagnosed PwP, as observed in the study by Mollenhauer et al. (2020).

Although *α*-syn aggregation and Lewy bodies are pathological hallmarks of PD, measuring α-syn in biofluids such as CSF or blood remains problematic due to the low specificity and sensitivity of current assays. This limited diagnostic performance has constrained the clinical utility of α-syn as a stand-alone biomarker (Roser et al., 2018). Recently, the α-syn seeding assay has shown high sensitivity and specificity (Siderowf et al., 2023). However, this assay requires a lumbar puncture and has not been shown to correlate with disease progression. Furthermore, this assay requires specialized equipment and training. A skin biopsy assay detecting phosphorylated α-syn has been developed, which also has good sensitivity and specificity (Gibbons et al., 2024). However, it requires a skin biopsy and has not been shown to correlate with disease progression. In addition, this assay requires specialized expertise for result interpretation.

While this study focused on miR-7-5p, dysregulation of other miRNAs is also likely involved in PD pathogenesis and could potentially be used along with miR-7-5p as progression biomarkers. Hoss et al. found that 125 miRNAs were different in the brains of PwP compared to HCs (Hoss et al., 2016). Other miRNAs thought to be involved in neuroinflammation in PD include miR-124, miR-195, miR-let-7a, miR-150, miR-330, miR-7116-5p, miR-190, miR-29c, and miR-30e (Shaheen et al., 2024). Using the PPMI dataset, Kern et al. found 34 miRNAs that changed over time in PwP (Kern et al., 2021). Unlike miR-7-5p, most of the other miRNAs had not been studied extensively in animal models of PD. Some of the miRNAs that have been studied in animal models include miR-19b-3p, which targets LRRK2, and miR-29c-3p, which inhibits the NLRP3 inflammasome. Of these, miR-7-5p is the only one that regulates alpha-synuclein expression and aggregation and inflammasome activity.

In a pre-clinical mouse model, miR-7-5p has been found to be possibly implicated in PD pathogenesis, supporting the idea that its decline could be part of the neurodegenerative process in PD. Furthermore, pesticides have been found to downregulate certain miRNAs, such as miR-7-5p (Li et al., 2019).

There is some discrepancy in the literature regarding whether miR-7-5p is increased or decreased in PwP compared to controls. Several studies have reported lower levels of miR-7-5p in PwP compared to HCs in whole blood (Kern et al., 2021), the substantia nigra (McMillan et al., 2017), the prefrontal cortex (Hoss et al., 2016), and serum (Zhou et al., 2016). However, other studies have found it to be increased in the CSF (Starhof et al., 2019), plasma (Ravanidis et al., 2020), serum (Citterio et al., 2023), and PBMCs (Alieva et al., 2015; Ardashirova et al., 2022). Notably, miR-7-5p was also elevated in blood extracellular vesicles (EVs) from idiopathic REM sleep behavior disorder (iRBD) patients who later converted to PD (Li et al., 2024). In contrast, EV miR-7-5p was found to be lower in iRBD patients compared to HCs. EV miRNAs are protected from ribonuclease degradation, which may make them a more reliable marker of disease than miRNAs from other sources, with less variability in results due to sample preparation. However, data remain limited, as these assays are not readily available or in use.

The reason miR-7-5p is not different between HCs and PwP at baseline needs to be further studied. It is possible that parts of the miR-7-5p regulatory network are intact early in the disease process, allowing the levels to normalize, and become dysregulated as the disease progresses. Circular RNAs are known to regulate miRNAs and may also become dysregulated later in the disease process. Future studies are needed to determine if parts of the miR-7-5p regulatory network are affected as the disease progresses.

In longitudinal studies, miR-7-5p levels in HCs have been observed to decline over time, possibly due to comorbid medical conditions associated with decreased miR-7-5p. miR-7-5p has been found to be downregulated in some cancers, so it is possible that some of the controls may have had underlying malignancies contributing to these changes (Dhara et al., 2025). As mentioned above, time in the study was no longer significant in HCs when comorbidities associated with decreased miR-7-5p were included in a model, including thyroid cancer, hypertension, atrial fibrillation, type 2 diabetes, and COVID-19 (Kim et al., 2018). In this model, there was a significant interaction between time in the study and comorbidities on miR-7-5p levels in HCs. There remained a significant correlation between miR-7-5p and time in the study in PwP when comorbidities were included in the model. There was no significant interaction between time in the study and comorbidities on miR-7-5p levels in this model. Previous studies have found that miR-7-5p does not correlate with baseline age in HCs (Brown et al., 2025; Dong et al., 2019; Heverhagen et al., 2018; Saeidi et al., 2023). Similarly, baseline age did not correlate with log[miR-7-5p] levels in HCs when sex was included in the model.

The main limitation of this study is that it included only drug-naïve PwP who were early in the disease process. However, the study benefits from a large longitudinal sample, and statistical analyses were designed to account for the effects of sex and age. In addition, the three-year longitudinal study period may not be sufficient to capture the critical window of activity loss for miR-7-5p in this population. Extending the study period to 5 or 10 years, as well as examining miR-7-5p levels in blood-derived EVs and cerebrospinal fluid, would likely provide a deeper understanding of its role in PD progression. Studying the population over a long period would also allow for a more accurate assessment of threshold changes that can be reliably detected between PwP and HCs and would help determine whether the relationship between miR-7-5p and disease progression is maintained later in the disease process. Another limitation of the study is that the association between miR-7-5p and NfL, as well as between miR-7-5p and clinical measurements, lost significance after time in the study was included in the model. This indicates that time plays a considerable role. However, we cannot entirely rule out the possibility that miR-7-5p and NfL are part of a shared neurodegenerative pathway without further mechanistic studies or models that can better disentangle these effects. As miR-7-5pis involved in inflammation and correlates with NfL, it could be a good biomarker both in the early and later stages of the disease process. It would be best to include miR-7-5p in a panel of biomarkers, such as NfL, to assess disease progression. However, this needs further investigation. In addition, the study should be replicated in a separate, large PD cohort, such as the Parkinson’s Disease Biomarkers Program (PDBP) cohort or the National Center for Excellence in Research on Parkinson’s Disease (NCER-PD) cohort, to ensure the validity of the results.

Based on animal models of PD, miR-7-5p could be implicated in PD pathogenesis. Its involvement is further supported by longitudinal decreases in its levels and by correlations with clinical measures of disease severity, as well as NfL.

## Conclusion

While miR-7-5p levels declined over time in PwP, it did not significantly decline over time in HCs when age, sex, and comorbidities associated with decreased miR-7-5p levels were considered. Furthermore, this study is the first to correlate miR-7-5p levels longitudinally with clinical severity and NfL levels. Our data highlight a modest association between whole blood miR-7-5p levels and PD progression. miR-7-5p may serve as a useful progression marker. However, further studies are needed, as the relationship between miR-7-5p, NfL, and clinical measures may reflect parallel processes, given the loss of significance when time was considered. These results are preliminary and require replication.

## Data Availability

The data analyzed in this study is subject to the following licenses/restrictions: in order to access PPMI data, there is an approval process based on the objectives of the proposed study. Requests to access these datasets should be directed to https://www.ppmi-info.org/access-data-specimens/download-data.
